# Surfactant Protein C-associated interstitial lung disease; three different phenotypes of the same *SFTPC* mutation

**DOI:** 10.1186/s13052-016-0235-x

**Published:** 2016-02-29

**Authors:** Teresa Salerno, Donatella Peca, Laura Menchini, Alessandra Schiavino, Renata Boldrini, Fulvio Esposito, Olivier Danhaive, Renato Cutrera

**Affiliations:** Pneumology Unit - Department of Pediatric Medicine, Bambino Gesù Children’s Hospital, IRCCS, Piazza S Onofrio 4, 00165 Rome, Italy; Research Laboratory, Bambino Gesù Children’s Hospital, IRCCS, Piazza S Onofrio 4, 00165 Rome, Italy; Department of Radiology, Bambino Gesù Children’s Hospital, IRCCS, Piazza S Onofrio 4, 00165 Rome, Italy; Department of Pathology, Bambino Gesù Children’s Hospital, IRCCS, Piazza S Onofrio 4, 00165 Rome, Italy; Pneumology Unit – Pediatric Hospital Santobono, Via Mario Fiore 6, 80123 Napoli, Italy; Department of Pediatrics, University of California San Francisco, 101 Potrero Avenue, San Francisco, CA 94110 USA; Department of Medical and Surgical Neonatology, Bambino Gesu’ Children’s Hospital, Piazza S.Onofrio 4, 00165 Rome, Italy

**Keywords:** Surfactant protein C, SP-C surfactant protein C gene, Children interstitial lung disease children

## Abstract

**Background:**

Monoallelic mutations of the Surfactant Protein C gene (SFTPC) are associated with Interstitial Lung Disease in children. I73T is the most common mutation, accounting for 30 % of all cases reported.

**Case presentation:**

We describe three patients carrying the same I73T SPC mutation with very different phenotypes, clinical course (ranging from mild respiratory symptoms to death for respiratory failure) and outcome.

**Conclusions:**

The disease mechanisms associated with SP-C mutations suggest that the combination of individual genetic background and environmental factors contribute largely to the wide variability of clinical expression. Infants, children and adults with ILD of unknown etiology should be investigated for SP-C genetic abnormalities.

## Background

The Surfactant Protein C gene (SFTPC, ENGS00000168484) encodes a 197 aminoacid apoprotein, subsequently cleaved into the mature Surfactant Protein C (SP-C), which is incorporated together with specific phospholipids and proteins into lamellar bodies in alveolar type 2 cells, then released via regulated exocytosis in the gas-liquid interface of the alveolus, playing a critical role in the modulation of lung mechanics. Lung diseases due to SFTPC mutations are inherited either as autosomal dominant trait with variable penetrance or as sporadic disease caused by a de novo single mutation, and were first described in 2001 [1]. These diseases have a broad spectrum of clinical presentations and manifestations, ranging from lethal neonatal respiratory distress syndrome to adult chronic interstitial lung disease (ILD). The cellular mechanisms underlying the pathogenesis of SFTPC mutation-associated ILD are not completely understood. Very few mutations affecting the mature peptide have been reported in human, most mutations being located in the C-terminus residue of the apoprotein. Mutations located in the BRICHOS domain flanking the C-terminus have been implicated in protein misfolding response and apoptosis. In fact, the proSP-C BRICHOS domain plays an essential chaperone role in preventing the highly hydrophobic mature SP-C peptide to aggregate in the endoplasmic reticulum and cause cell death [2, 3]. Conversely, non-BRICHOS mutations in the C-terminus linker domain (I73T, E66K) interfere with autophagic vacuole formation and maturation, causing a distinct ultrastructural phenotype with large intracytoplasmic vacuoles containing unstructured phospholipids [4, 5]. The g.1286 T > C mutation, leading to a substitution of threonine to isoleucine at codon 73 (I73T), is now recognized as the most common SP-C mutation, accounting for about 30 % of all cases reported [6–9]. We describe three patients with different clinical presentation, whose common denominator is the I73T mutation.

## Case presentation

Case #1 is a 3 years old term-born girl referred for persistent cough and dyspnea and intermittently required oxygen supplementation started one month before presentation, and chest CT performed 3 months after onset showed diffuse ground glass opacities and subpleural parenchymal micronodules. She is the second daughter of non-consanguineous healthy parents. Her sister died in the first year with respiratory insufficiency of unidentified etiology. The child was in good general condition. Chest auscultation was unremarkable. Humoral and cellular immunity testing, sweat tests and genetic analysis for cystic fibrosis,, cardiac evaluation, abdominal ultrasound all resulted normal. CT scan confirmed widespread patchy ground glass opacities and subpleural micronodules, with interstitial thickening. Bronchoscopy showed normal airways; bronchoalveolar Lavage (BAL) showed normal cytology and lipid index, negative bacterial culture, and negative respiratory viral immunofluorescence and PCR tests. SFTPC gene sequencing detected the I73T mutation. Parents, who were both asymptomatic, were not mutation carriers. The genetic findings are difficult to interpret, as parental testing suggests a de novo mutation, but would imply that the sibling died of an unrelated cause. The child was started on hydroxychloroquine 5 mg/kg/day per oral (HCQ). Her cough and dyspnea improved after one week and she was discharged home on room air. Three months after she was still completely asymptomatic, and a repeat chest CT was improved, showing disappearance of the ground glass abnormalities but persistence of micronodules. After six months we observed a complete recovery of the CT findings. At 3 years of age the child is still on treatment, with no respiratory symptoms.

Case #2 is a 4 years old male born at term, asymptomatic with normal growth and development until he presented at 6 months of age with a respiratory syncytial virus (RSV) bronchiolitis, which evolved in acute respiratory distress syndrome (ARDS) requiring surfactant administration and mechanical ventilation for one week. Bronchoscopy was normal; lung CT showed ground-glass attenuations suggestive of ILD. Immunological screening showed a mild IgA and IgG deficiency. Sweat test and cystic fibrosis genetic test were normal. The infant was discharged home after one month, on oxygen and oral methylprednisolone. He was referred to our center at 12 months,of age. The infant was in poor nutritional status, with a weight <10^th^ percentile. Clinically, he had intercostal retractions, diffuse crackles on auscultation and hypoxemia on room air. Infection workup was negative. Bronchoscopy showed no airway anomalies but lipid-laden alveolar macrophages were seen on BAL fluid examination, indicating chronic tracheal aspirations. CT scan showed widespread ground glass opacities and multiple subpleural and intraparenchymal cystic areas predominantly in the upper lobes. The infant continued oxygen and steroids at higher dosage, but a repeat chest CT at 18 months showed more extensive anomalies. An open lung biopsy was then performed, and showed thickened interstitium with inflammatory cells and fibrosis, alveolar type II cell hyperplasia, and cellular and amorphous material in alveolar lumen, a picture consistent with desquamative interstitial pneumonia (DIP), suggestive of genetic surfactant disorders. SFTPC gene sequencing revealed a mono-allelic I73T mutation. Parents declined genetic testing. The infant was discharged with monthly methylprednisolone pulse therapy plus oxygen. Despite an initial improvement, the repeat chest CT after 10 pulses showed progression of ILD. At that stage, HCQ was added to methylprednisolone. After 6 months we observed an improvement in SaO2/CO2 nocturnal monitoring, allowing weaning then discontinuation of oxygen therapy and switching to hydrocortisone as a lower potency steroid regimen. At the age of 36 months, since chest CT was stable compared to the previous one, HCQ was replaced by azithromycin given its better safety profile. At 4 years of age, chest CT was unchanged and the infant was stable off oxygen and steroids.

Case #3, male, born at term with no neonatal issues. Family history was unremarkable. The infant had several episodes of lower respiratory tract infections starting at 2 months of age. At one year of age, he was hospitalized in the pediatric intensive care unit for acute respiratory failure, intubated and ventilated. Cystic fibrosis and immunodeficiency were excluded. Chest radiography showed diffuse opacities of both lung fields and interstitial thickening. Lung CT showed multiple ground-glass consolidations, and thickening of the bronchial vessels. He underwent a lung biopsy, which showed DIP. Immunohistochemistry showed marked proSP-C accumulation in hyperplastic type 2 cells and in alveolar macrophages. Electron microscopy documented abnormal type II pneumocytes with numerous large cytoplasmic vesicles (endosomes) filled with unstructured phospholipids, consistent with SP-C deficiency [5, 10]. Molecular analysis identified an I73T mutation, inherited from mother who is an asymptomatic carrier. HCQ was added to steroids after the report of the biopsy but the patient died after 18 days of intractable respiratory failure.

ILD in infants have a wide range of etiologies including genetic abnormalities of the surfactant system, immunological defects, environmental exposures and idiopathic entities such as neuroendocrine cell hyperplasia of infancy and pulmonary interstitial glycogenosis [11]. These three cases illustrate the variability of clinical severity associated with the I73T mutation: one asymptomatic at 3 years, one with chronic lung disease at 4, and one who died at 1. Similar phenotypic variability was described in a single Mauritanian family affected with I73T, where 5 affected subjects had very different onset, symptoms, radiological presentation and outcomes [12]. The underlying mechanisms of this variable penetrance are not fully understood.

Trans-heterozygosity has been reported as a molecular mechanism explaining this expression variability. In a recently published report, three infants with SP-C deficiency due to an I73T mutation and unusually early respiratory symptoms carried an additional mutation in the ATP binding cassette protein A3 gene (ABCA3), a phospholipid carrier essential for lamellar body formation and surfactant intracellular assembly. ABCA3 deficiency, which typically leads to lethal neonatal-onset respiratory distress syndrome, is transmitted in autosomal recessive mode and heterozygous carriers are unaffected [13]. However, this report suggests that mono-allelic mutations in different genes, which would be asymptomatic if isolated, may inflict multiple hits may have cumulative effects on the surfactant synthesis pathway and modify the disease course. We screened the 3 patients in this report for mutations in the SFTPB, ABCA3 and NKX2.1 genes, all associated with lung disease in human, and none were found.

There are several lines of evidence that respiratory infection, particularly RSV, can trigger severe lung injury and catastrophic respiratory failure in SP-C mutation carriers. Genetically modified animal overexpressing a common human mutant pro-SP-C (Δexon4, a BRICHOS domain mutation) show an increased rate of alveolar cell death [14]. SP-C deficient mice exposed to RSV have a stronger inflammatory response and more severe interstitial lung disease than controls, and restoration of SP-C expression by transgene therapy has a rescue effect in these animals, which could pave the way to future gene therapy in human [15, 16]. SP-C mutations result in loss of TLR3 modulation, IL-8 induction and paracrine modulation of the CCR2 and CXCR1 chemokine receptors on leukocytes leading to uncontrolled inflammation and fibroblast proliferation. In a recent clinical study by our group, 4 out of 7 cases were revealed by an unusually severe/protracted bronchiolitis episode, and similar observation was reported by others [5, 7]. In this report, cases #2 and 3 confirm this concept.

There have been no controlled trials of any treatment in children ILD and the management is based upon uncontrolled studies, case series, case reports and unsystematic observations. Steroids, hydroxychloroquine and azithromycin are commonly used in SP-C-related ILD. Azithromycin has anti-amyloid properties, and hydroxychloroquine affects intracellular proSP-C processing; hence these drugs may possibly have selective effects on specific mutations, although there are currently no clinical data available to support this concept. Lung transplantation is an option for children with end-stage ILD, but its use is limited by donor availability and poor outcomes [11, 17, 18].

The broader availability of genetic testing has made early, noninvasive diagnosis accessible to clinicians. CT findings were consistent with ILD in the three patients, consistently with recent literature [19]. With proper technique, chest CT imaging patterns may sufficiently suggest a diagnosis so as to avoid the need for surgical lung biopsy. Chest CT is also useful in monitoring the effects of treatment, as there is a strong correlation between clinical improvement and CT findings trend: as previously described, even in our patients 1 and 2, clinical improvement and weaning-off oxygen were associated with a reduction of ground glass opacities.

When lung biopsy is needed, chest CT is important in guiding the selection of biopsy sites. Surgical lung biopsy using video-assisted thoracoscopy (VATS) is recommended for infants with clinical urgency to identify the specific form of ILD or in whom other diagnostic evaluations have not yielded a specific diagnosis. Lung biopsy specimens should be handled according to established protocols, with sections available for histopathology, immunohistochemistry, microbiologic culture, electron microscopy, immunofluorescence or other special studies, in consultation with a pediatric pathologist experienced in childhood ILD [13].

## Conclusions

This report highlights the importance of screening for SFTPC mutation in children with ILD of unknown etiology. The clinical variability associated to the I73T mutation demonstrates the importance of disease-modifying such as respiratory tract infection, particularly RSV. Infants with SP-C mutations should benefit from early prophylaxis measures, such as palivizumab. There is a great need for multicenter randomized controlled trials in order to determine the best regimens for existing drugs that seem to improve the course of the disease, as well as additional basic and translational research to identify and test newer agents that improve long-term prognosis.

Ethics approval and consent to participate: Written consent was obtained from the parents for publications of these case reports. A copy of the written consent is available for review by the Editor-in-Chief of this journal.

## Abbreviations

BAL: bronchoalveolar lavage
CFTR: cystic fibrosis transmembrane regulator
HCQ: hydroxychloroquine
ILD: interstitial lung disease
SP-C: surfactant protein C
SFTPC: surfactant protein C gene
